# The Role of Alveolar Epithelium in Radiation-Induced Lung Injury

**DOI:** 10.1371/journal.pone.0053628

**Published:** 2013-01-11

**Authors:** Celine Almeida, Devipriya Nagarajan, Jian Tian, Sofia Walder Leal, Kenneth Wheeler, Michael Munley, William Blackstock, Weiling Zhao

**Affiliations:** 1 Department of Radiation Oncology, Wake Forest School of Medicine, Winston-Salem, North Carolina, United States of America; 2 Department of Radiology, Wake Forest School of Medicine, Winston-Salem, North Carolina, United States of America; 3 Brain Tumor Center of Wake Forest University, Wake Forest School of Medicine, Winston-Salem, North Carolina, United States of America; 4 Radiation Research Laboratories, Department of Radiation Medicine, Loma Linda University and Medical Center, Loma Linda, California, United States of America; National Cancer Institute, United States of America

## Abstract

Pneumonitis and fibrosis are major lung complications of irradiating thoracic malignancies. In the current study, we determined the effect of thoracic irradiation on the lungs of FVB/N mice. Survival data showed a dose-dependent increase in morbidity following thoracic irradiation with single (11–13 Gy) and fractionated doses (24–36 Gy) of ^137^Cs γ-rays. Histological examination showed a thickening of vessel walls, accumulation of inflammatory cells, collagen deposition, and regional fibrosis in the lungs 14 weeks after a single 12 Gy dose and a fractionated 30 Gy dose; this damage was also seen 5 months after a fractionated 24 Gy dose. After both single and fractionated doses, i] aquaporin-5 was markedly decreased, ii] E-cadherin was reduced and iii] prosurfactant Protein C (pro-SP-c), the number of pro-SP-c^+^ cells and vimentin expression were increased in the lungs. Immunofluorescence analysis revealed co-localization of pro-SP-c and α-smooth muscle actin in the alveoli after a single dose of 12 Gy. These data suggest that, i] the FVB/N mouse strain is sensitive to thoracic radiation ii] aquaporin-5, E-cadherin, and pro-SP-c may serve as sensitive indicators of radiation-induced lung injury; and iii] the epithelial-to-mesenchymal transition may play an important role in the development of radiation-induced lung fibrosis.

## Introduction

Thoracic radiotherapy (RT) is one of the important therapeutic modalities for treating lung cancer, breast cancer, and various lymphomas. Pneumonitis and lung fibrosis are the major radiation-induced complications following thoracic RT [1], [2]. Symptoms of radiation pneumonitis, such as low-grade fever, mild cough, or dyspnea, usually occur 2–3 months after irradiation. The risk of developing radiation-induced pneumonitis depends on the dose, volume of lung irradiated, and dose fractionation scheme [1], [2]. Radiation can disrupt epithelial and endothelial integrity leading to edema, recruitment of leukocytes, angiogenesis, and a cascade of molecular events that alters the microenvironment [3] that creates a self-sustaining cycle of inflammation and chronic oxidative stress [3], [4]. The main histological features of radiation-induced pneumonitis include, i] a dose-dependent leakage of proteins into the alveolar space, ii] thickening of the alveolar septa, iii] edema of the interstitium, iv] alteration of the capillaries, and v] changes in type II pneumocytes and alveolar macrophages [5]. Lung fibrosis can appear 6 months to a year after irradiation, and is characterized by excessive fibroblast proliferation and massive deposition of extracellular matrix [6]. The alveoli eventually collapse and are obliterated by connective tissue. Patients with radiation-induced lung fibrosis have severe physiologic abnormalities and chronic respiratory failure [7]. Although inflammation and fibroblast/myofibroblast activation have been recognized as important contributors, the exact mechanism(s) underlying pulmonary fibrosis remain elusive [3].

Respiratory epithelium performs an important function as a barrier against microbial infection/exterior insults and modulation of the airway immune response [8]. Severe injury and retarded repair of the alveolar epithelium is sufficient to promote the fibrotic process [9], [10]. The alveolar epithelium consists of two cell types, alveolar epithelial type I (AE1) and alveolar epithelial type II (AE2) cells. AE1 cells are responsible for gas exchange, regulate liquid homeostasis in the alveoli, and are highly susceptible to oxidative stress and toxic insults. AE2 cells serve two major functions in the lung, i] synthesizing, secreting, and regulating lung surfactants, and ii] repopulating AE1 cells [8]. Recent studies suggest that epithelial cells can also undergo transdifferentiation into myofibroblasts, through a process termed “epithelial–mesenchymal transition” (EMT) [11]. Alveolar EMT has been demonstrated in human idiopathic pulmonary and experimental pulmonary fibrosis [12], [13]. EMT is characterized by, i] a shift from an epithelial to a bipolar morphology, ii] increased expression of the mesenchymal markers, alpha-smooth muscle actin (α-SMA) and vimentin, and iii] decreased expression of the epithelial marker, E-cadherin [14]. Although a growing body of evidence supports the involvement of EMT in the development of fibrosis, its role in radiation-induced lung injury has not been established *in vivo*.

The sensitivity of the mouse lung to radiation-induced pneumonitis and fibrosis is strain-dependent [15]–[17]. Sharplin et al. compared lung injury in two C3H, three C57BL and one CBA mouse strain (dose range studied was 10–14.5 Gy). C3H and CBA strains developed only classical pneumonitis during the early and late phase; no visible fibrosis was observed. However, C57BL mouse strains developed extensive fibrosis during the latent period [18] Radiation-induced fibrosis in C57BL strain occurs following whole-thoracic irradiation (WTI) with single doses of 12–15 Gy as early as 24 weeks postirradiation [19]. In contrast, focal alveolar fibrosis is not observed until 66 weeks after 30 Gy of fractionated (f) WTI [19], [20]. The translatability of the C57BL/6 mouse data to humans has been questioned recently in an NIH sponsored workshop on Animal Models and Medical Countermeasures Development for Radiation-Induced Lung Damage [21]. The primary concerns with the C57BL/6 model were that, i] overt pneumonitis is poorly defined in the C57BL/6 mouse, ii] decrements in lung function and ultimately the survival of these mice after irradiation is predominantly due to an accumulation of fluid in the pleural space (pleural effusion) which is not related to either radiation-induced pneumonitis or fibrosis [22], [23]. Studies have been focused on development of new mouse models for assessing radiation-induced chronic lung injury [22], [23]. Recently, FVB transgenic mice have been used to study the effect of various genes on radiation-induced organ injury and tumor growth [24]–[26]; however, the lung radiosensitivity of FVB mice has not been studied. Here, we report the response of female FVB/N mice to single and fractionated doses of WTI. Our data suggest that in female FVB/N mice, i] the lungs are sensitive to radiation, ii] WTI modulates the alveolar epithelium, and iii] WTI induces EMT in the lung.

## Materials and Methods

### Animals

Six week-old female FVB/N were purchased from The Jackson Laboratory (Bar Harbor, Maine). The mice were housed in the AALAC-accredited animal care facility at Wake Forest School of Medicine (WFSM) and acclimated for 2 weeks before initiating the studies. All animal care and experimental procedures were performed in strict accordance with the NIH Guide for Care and Use of Laboratory Animals. All protocols were approved by the WFSM Institutional Animal Care and Use Committee before experiments began.

### Irradiation

Prior to irradiation, 3 mice were CT scanned to determine the size of the lungs, and a Cerrobend collimator was constructed to restrict the beam to just the thorax. Mice were irradiated using a ^137^Cs irradiator (Model 81A, Shephend & Associates, Glendale, CA) at a dose rate of 3.68 Gy/min. Groups of mice ( n = 10 ) received single (11, 12, or 13 Gy) or fractionated (24, 30 or 36 Gy given as 3, 4, 5 or 6 Gy fractions, 2 fractions/wk over 3 wk) dose of thoracic irradiation. Animals were monitored up to 5 months postirradiation, and the body weight and survival of animals recorded.

### Sample collection

The surviving mice in the 12 Gy single dose group and in the 30 Gy fractionated dose group were euthanized at 14 weeks with ketamine/xylazine (80/20 mg/kg, i.p.) due to stress symptoms. The mice treated 24 Gy fractionated dose were euthanized at 1, 2 and 5 months postirradiation. The left lung was inflated with PBS, dissected and fixed in 10% neutral formalin for histological and immunohistochemical analysis. The right lungs were snap frozen in liquid nitrogen and kept at −80°C for protein analysis.

### Histopathology

Lung sections (5 µm) were stained with either hemotoxylin/eosin (H&E) to assess the histological changes or with Masson's trichrome to identify the sites of collagen deposition. All analyses were performed on coded slides by a blinded pathologist (JT).

### Immunohistochemical analysis

Lung sections (5 µm) were mounted on slides, deparaffinized, and hydrated in xylene, 95% ethanol, 80% ethanol, 75% ethanol, and 1X PBS, pH 7.4. Following antigen retrieval with citrate buffer, tissue sections were preincubated with Rodent Block M for 20 min (Biocare Medical, Concord, CA) and then incubated with pro-surfactant protein-c (pro-SP-c, 1∶2,000) , CD 45 or α-SMA (1∶500) antibodies (Abcam, Cambridge, MA). Control slides were incubated with the appropriate IgG. After washing with PBS, the tissue sections were incubated with rabbit or mouse HRP-polymers for 30 min (Biocare Medical, Concord, CA); diaminobenzidine (DAB) was used for visualization. The number of prosurfactant Protein C (pro-SP-c) positive cells was counted using morphormetric method.

### Double immunofluorescence staining

A double-color immunofluorescence analysis was performed to identify the expression of α-SMA, a mesenchymal cell marker, in AE2 cells in the lung. Briefly, after deparaffinization, antigen retrieval, and incubation with Rodent Block M as described above, the sections were incubated with a mixture of anti-α-SMA (1∶500, Abcam, Cambridge, MA) and anti-pro-SP-c (1∶2,000, Abcam, Cambridge, MA) antibodies at 4°C overnight. After washing with PBS, the sections were incubated with FITC conjugated Alexa Fluor 488 goat anti-mouse (Invitrogen, Carlsbad, CA) and Texas Red-conjugated anti-rabbit secondary antibodies (Abcam) at room temperature for 30 min. Nuclei were counterstained with 4′-6-diamidino-2-phenylindole (DAPI), and the sections analyzed using a fluorescence microscope.

### Western Blot analysis

Frozen lung tissue (50 mg) was ground in a mortar with liquid nitrogen, homogenized with 1 mL RIPA buffer with 1 m*M* PMSF, 1 µg/mL aprotinin, 1 µg/mL leupeptin, 1 m*M* Na_3_VO_4_ and 1 m*M* NaF, and stored in aliquots at −70°C until assayed. The lysate (20 µg) was mixed with an equal volume of sample buffer, denatured by boiling, and then separated on a 10–15% polyacrylamide mini-gel. The proteins were transferred to nitrocellulose membranes (Amersham, Arlington Heights, IL), blocked with 5% milk, and incubated overnight with pro-SP-c (1∶2,000, Abcam), TGF-ß1 (1∶1,000, Abcam), vascular cell adhesion molecule 1 (Vcam-1) (1∶1,000 , Santa CruZ, Biotechnology, Santa Cruz, CA), E-cadherin (1∶1,500, Abcam), vimentin (1∶1,000, Santa Cruz Biotechnology), aquaporin-5 (1∶1,000, Santa Cruz Biotechnology) or ß-actin antibodies (1∶10,000, Sigma, St. Louis, MO). The blots were then incubated with anti-mouse or anti-rabbit IgG horseradish peroxidase conjugated antibodies (GE healthcare, Piscataway, NJ) for 1 h at room temperature. Finally, the signal was detected using standard chemical luminescence methodology (ECL plus; GE healthcare). For densitometrical analysis of bands on western blot, the densitometric signal for the targeting protein (for example E-cadherin) was normalized to ß-actin and expressed as the density ratio of target to non-irradiated control.

### Morphometric analysis

The morphometric analysis of pro-SP-C^+^ cell number was conducted using stereological Investigator software as previous described with modification [27], [28]. Ten fields (250 µm×250 µm) on the stained slide were randomly selected. Images were acquired from each field and the number of cells with positive staining counted in the selected fields and expressed as the number of cells/mm^2^. Large airways and lung vessels were excluded.

### Statistical Analysis

Statistical analysis was performed using one-sample Student's t tests to compare the differences between the irradiated and unirradiated lung tissue response. A *p* value of ≤0.05 was considered significant.

## Results

### Survival after single and fractionated doses of WTI

**Figure 1:**
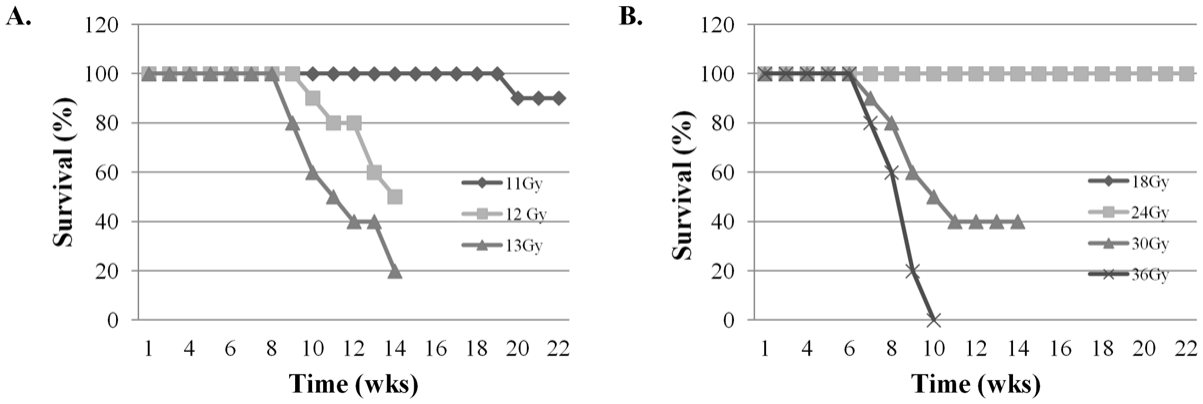
Irradiating the lungs of young adult female FVB/N mice with single and fractionated doses of ^137^Cs γ-rays leads to dose-dependent increases in mortality. Groups of 8–10 week-old female FVB/N mice were irradiated with single (11–13 Gy) or fractionated (18–36 Gy) doses of γ rays, and their survival recorded up to 22 weeks postirradiation.

To determine the sensitivity of FVB/N mice to WTI, 8–12 week-old female mice were irradiated with single (11–13 Gy) or fractionated doses (18–36 Gy) of WTI. Single doses of WTI led to a dose-dependent decrease in survival (Fig. 1A); ∼50% of the mice were dead at 11 and 14 weeks after irradiation with 13 and 12 Gy, respectively. In contrast, 90% of the mice irradiated with 11 Gy survived to 5 months postirradiation. Similarly, fWTI led to a dose-dependent decrease in survival (Fig. 1B); ∼50% of mice were dead at 9 and 11 weeks after irradiation with total doses of 36 and 30 Gy, respectively. In contrast, all of the mice irradiated with total doses of 24 Gy survived up to 5 months postirradiation. The biological effective dose (BED) for an 11 Gy is about 51.33 and 56 for 24 Gy fractionated dose. The BED for 12 Gy is roughly an equivalence of 36 Gy fractionated dose. However, our observation on survival rates showed that biological effect of single dose appeared more toxic than fractionated dose.

### Histological changes after single and fractionated doses of WTI

**Figure 2:**
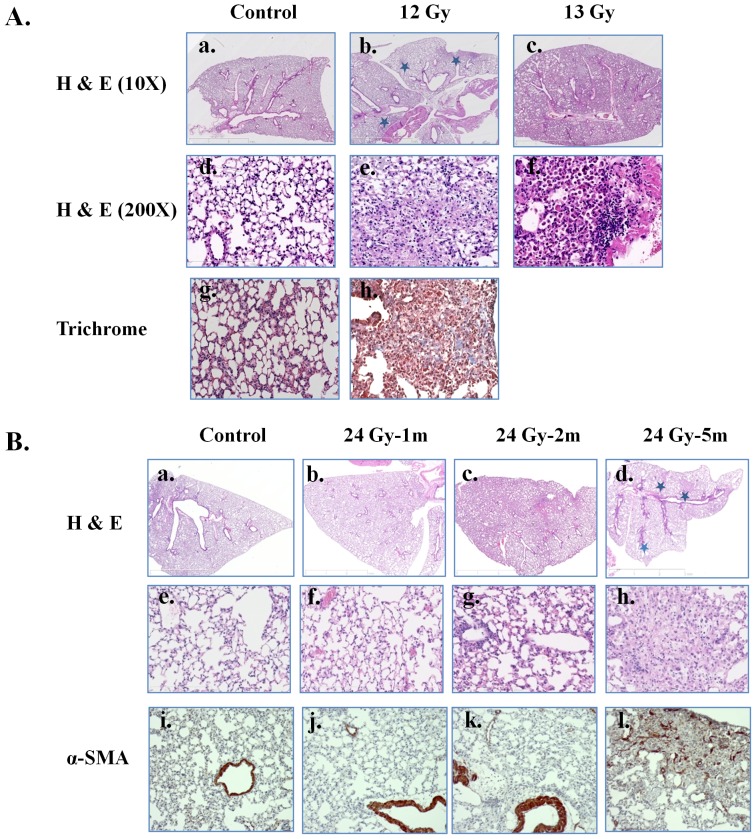
Irradiating the lungs of young adult female FVB/N mice with single and fractionated doses leads to regional fibrosis and collagen deposition and increased inflammatory cell infiltration. Female FVB/N mice were received thoracic radiation with 12 and 13 Gy single dose or fractionated dose 24 Gy. Mice were euthanized at 14 weeks (12 and 13 Gy groups) or 1, 2 and 5 months (24 Gy fractionated group) postirradiation, respectively. Lung sections were stained with H & E, Massons Trichrome and/or α-SMA. A shows representative lung images of H & E staining (control: a and d; irradiated with a single 12 Gy: b and e; 13 Gy: c and f) and Masson's trichrome staining (control: g; 12 Gy: h) for mouse treated with a single dose radiation. B shows representative lung images of H & E staining (control: a and d; 1 m postirradiation (PI) with 24 Gy fWTI: b and f; 2 m PI: c and g; 5 m PI: d and h) and α-SMA staining (control: i; 1 m PI: j; 2 m PI: k; 5 m PI: l) for mice treated with 24 Gy dose of fWTI.

The surviving mice irradiated with a single dose of 12 Gy or a fractionated dose of 30 Gy showed stress symptoms at 14weeks postirradiation. Consequently, they were euthanized, the lungs excised, and a portion of the lung tissue prepared for histological analysis after H & E or Masson's trichrome staining. After a single 13 Gy dose, H & E stained lungs showed markedly thickened alveolar walls, collapsed alveoli, foam-like cells in the alveolar space, and diffuse accumulation of inflammatory cells (Fig. 2). Similar histological changes were noted in the lungs of mice receiving 30 Gy fWTI (data not shown). Increased inflammatory cell infiltration, collagen deposition, and regional fibrosis were seen in the mouse lungs irradiated with a single dose of 12 Gy (Fig. 2A, middle panel). No markedly chances in lung morphology were observed at 1 month postirradiation with 24 Gy fWTI, when compared with the non-irradiated control (Fig. 2B, second panels). By 2 months postirradiation, enhanced inflammatory cell infiltration and alveolar wall thickness have been seen in the irradiated lungs (Fig. 2B; third panels). At 5 months postirradiation, multiple fibrotic lesions were presented in the irradiated lungs (Fig. 2B, right panel). Immunohistochemical staining with α-SMA, a marker of smooth muscle cells/myofibroblasts, showed increased accumulation of myofibroblasts in the these lungs (Fig. 2B, lower panels).

### WTI leads to dose- and time-dependent reductions in aquaporin-5

**Figure 3:**
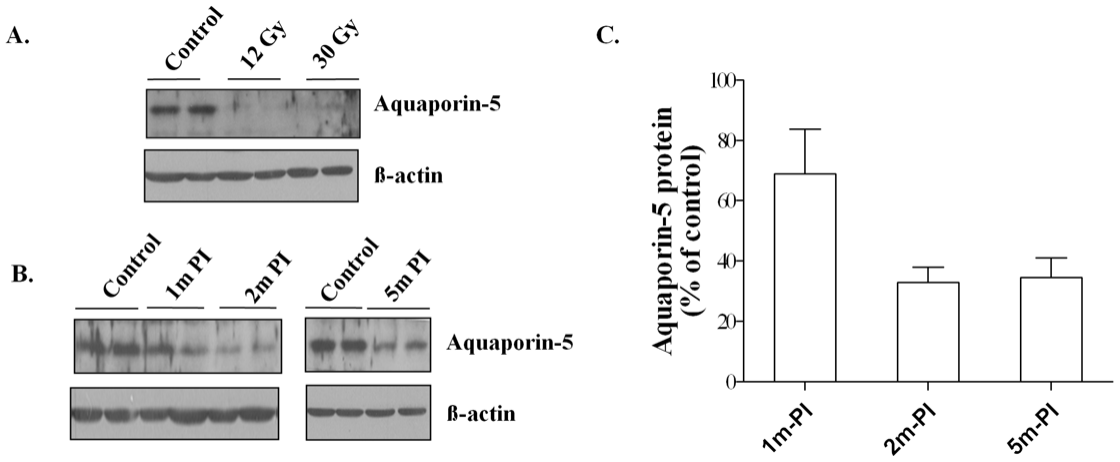
Irradiating the lungs of young adult female FVB/N mice with either a single 12 Gy dose or fractionated 24 or 30 Gy doses of ^137^Cs γ-rays leads to a marked decrease in the expression of aquaporin-5 in the lung. Lung tissues were collected at 14 weeks (12 Gy and 30 Gy) or at 1, 2 and 5 months (24 Gy) PI. Cell lysates were generated from the snap-frozen left lung from 4 mice and run on a 10–15% polyacrylamide mini-gel. Each lane contains the pooled lysate from 2 mice. ß-actin served as loading control. **A**: The reduction in aquaporin-5 protein at 14 weeks after a single 12 Gy or a fractionated 30 Gy dose is similar. **B**: The reduction in aquaporin-5 protein occurs as early as 1 month after a fractionated 24 Gy dose and remains reduced for the next 4 months. **C**: Quantification of the aquaporin-5 protein levels in 3B shows that the reduction in aquaporin-5 is progressive over the first 2 months postirradiation (PI) and then remains at a relatively constant low level up to 5 months PI. Densitometry was used to quantify the protein in each lane; all irradiation values in 2C were normalized to the unirradiated control values obtained on the same gel. The data in 2C are the mean ± 1 SEM.

Aquaporin-5 is an important AE1 cell-specific water channel protein that mediates water transport across the airway epithelium [29]. To examine the effect of radiation, the protein level of aquaporin-5 in irradiated and non-irradiated lung tissues was determined using Western blots. At 14 weeks postirradiation, a similar marked reduction in aquaporin-5 was measured in lungs irradiated with a single 12 Gy dose or a 30 Gy dose of fWTI (Fig. 3A). A time-dependent reduction in the aquaporin-5 level was also measured in lungs irradiated with 24 Gy fWTI (Fig. 3B). Densitometric analysis revealed that the mean level of aquaporin-5 in the irradiated lungs was reduced by 32, 67, and 66% at 1, 2 and 5 months postirradiation when compared with unirradiated controls (Fig. 3C).

### WTI leads to an increase in pro-SP-c protein and pro-SP-c^+^ AE2 cells

**Figure 4:**
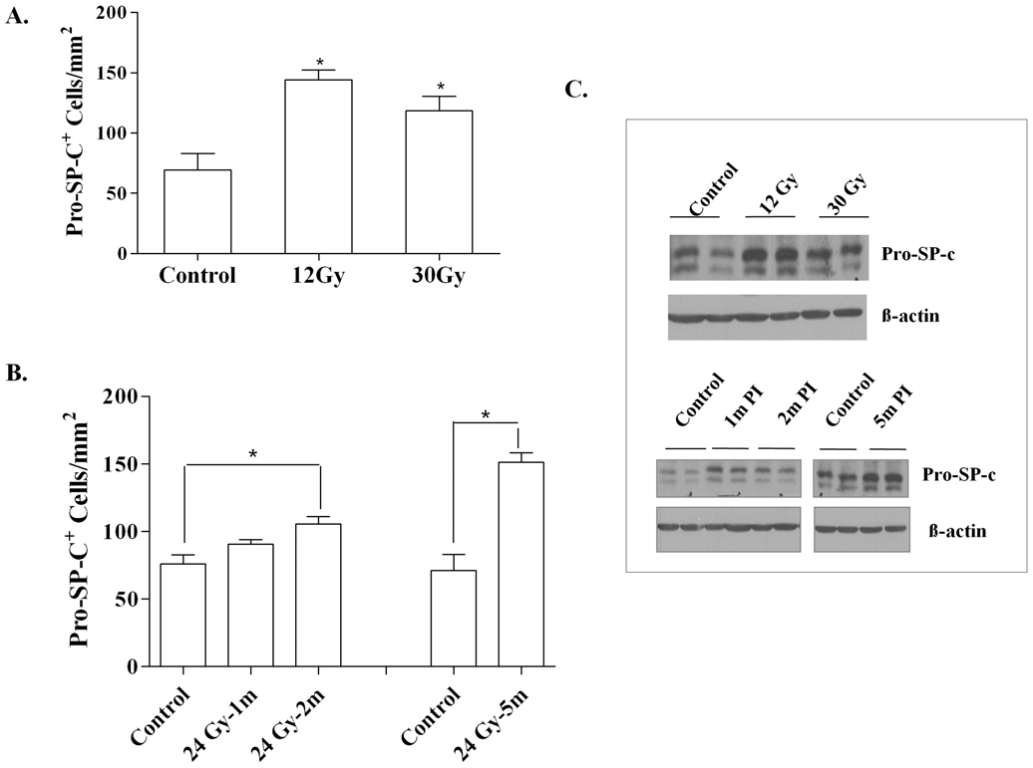
Irradiating the lungs of young adult female FVB/N mice with either a single 12 Gy dose or fractionated 24 or 30 Gy doses of ^137^Cs γ-rays leads to increases in pro-SP-c protein and pro-SP-c positive cells in the lung. **A**: The increase in pro-SP-c^+^ cells is similar at 14 weeks after a single 12 Gy dose or a fractionated 30 Gy dose. **B**: The increase in pro-SP-c^+^ cells after a fractionated 24 Gy dose is progressive over the first 2 months postirradiation (PI) and then remains relatively constant up to 5 months PI. **C**: Upper gel, the increase in pro-SP-c protein at 14 weeks after a single 12 Gy or a fractionated 30 Gy dose is similar. Lower gel, pro-SP-c protein is increase by 1 month PI and remains relatively constant up to 5 months PI. For 4A and 4B, lung tissues were collected and prepared for histochemical analysis either at 14 weeks (12 Gy and 30 Gy) or 1, 2 and 5 months (24 Gy) PI. Sections (5 µm) of lung tissue were stained with pro-SP-c antibodies, and the pro-SP-c^+^ cells were analyzed using morphometric method. Data are the mean ± 1 SEM; n = 3; ^*^ *p*<0.05 vs. sham control. The gels in 4C were prepared and analyzed as described in Fig. 3.

A similar increase in the number of pro-SP-c^+^ AE2 cells was measured in the lungs irradiated with a single 12 Gy dose or a 30 Gy dose of fWTI (Fig. 4A). After 24 Gy of fWTI, the number of pro-SP-c^+^ cells increased up to 2 months postirradiation when compared with unirradiated controls and remained relatively constant over the next 3 months (Fig. 4B). The pro-SP-c protein level was increased similarly at 14 weeks after a single 12 Gy dose and 30 Gy fWTI; the time-dependence of the increase in the pro-SP-c protein levels (Fig. 4C) was similar to that seen for the increase in pro-SP-c^+^ cells (Fig. 4B) after 24 Gy fWTI.

### WTI differentially modulates E-cadherin and vimentin protein levels

**Figure 5:**
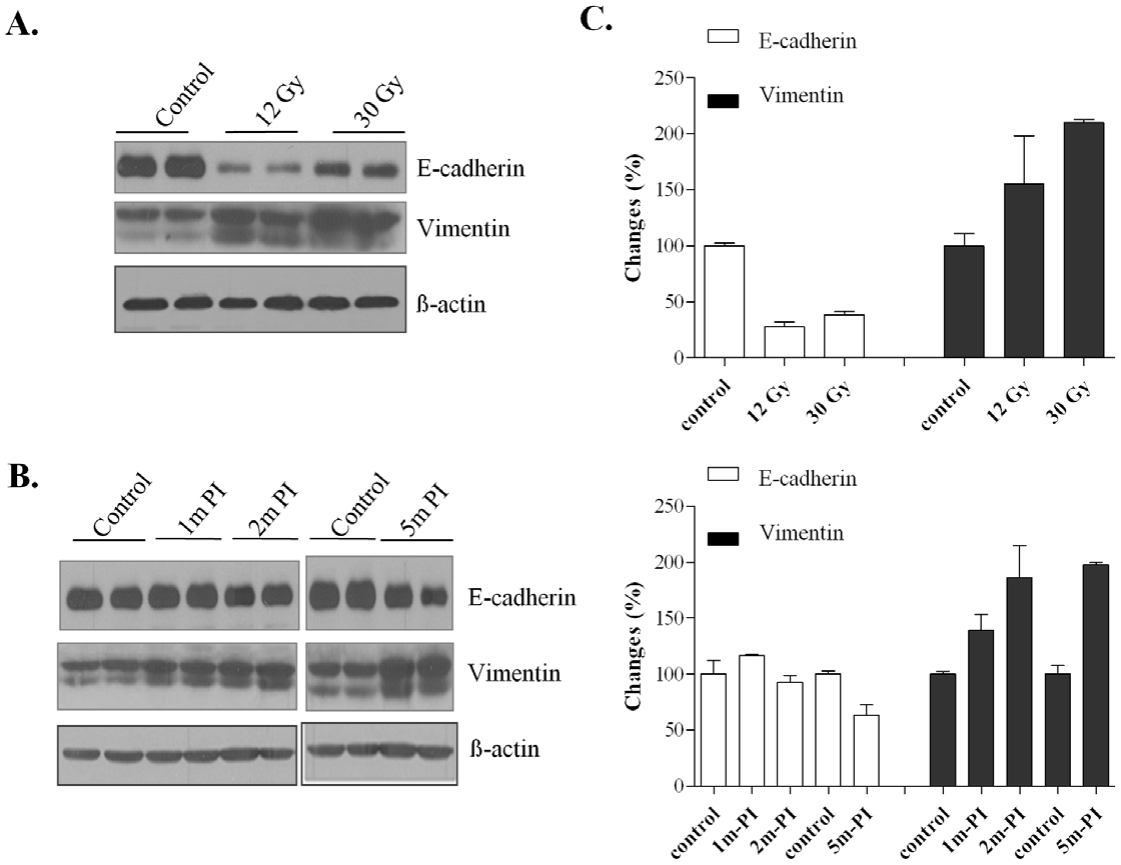
Irradiating the lungs of young adult female FVB/N mice with either a single 12 Gy dose or fractionated 24 or 30 Gy doses of ^137^Cs γ-rays leads to decreased levels of E-cadherin and increased levels of vimentin protein in the lung. **A, C**: Although the vimentin data is variable, the decrease in E-cadherin and the increase in vimentin are similar at 14 weeks after a single 12 Gy dose or a fractionated 30 Gy dose. **B, D**: After a fractionated 24 Gy dose, there is a slight increase in E-cadherin at 1 month postirradiation (PI) followed by a progressive decrease up to 5 months PI. After a fractionated 24 Gy dose, there is a progressive increase in vimentin up to 2 month PI that remains relatively constant for the next 3 months. All methods and analyses are identical to those described in Fig. 3.

**Figure 6:**
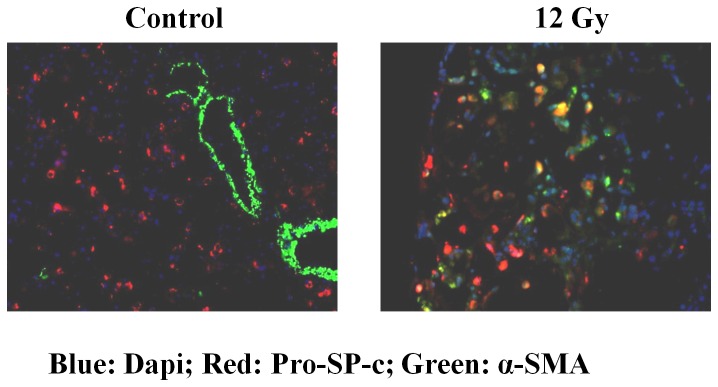
Pro-SP-c and α-SMA protein co-localize in the lungs of FVB/N mice at 14 weeks postirradiation with a single dose of 12 Gy. Lung section (5 µm) from an unirradiated control (A) and an irradiated mouse (B) were co-stained with pro-SP-c and α-SMA antibodies. In the representative photomicrographs the pro-SP-c protein is red, the α-SMA protein is green, and the nuclei are blue.

E-cadherin is a calcium-dependent adhesion molecule expressed on epithelial cells [30]. Normal expression and functional activity of E-cadherin are critical for the maintenance of tight junctions between epithelial cells and for maintaining normal function of the paracellular barrier in the airway epithelia. A 74% and 62% reduction in the level of E-cadherin protein was measured in the lungs 14 weeks after a single 12 Gy dose or a 30 Gy dose of fWTI, respectively (Fig. 5A, C). After 24 Gy fWTI, E-cadherin protein levels increased slightly at 1 month and then decreased by 40% over the next 4 months postirradiation (Fig. 5B, C). In contrast, the protein level of vimentin, a mesenchymal marker, increased ∼2 fold at 14 weeks after irradiation with a single 12 Gy dose or 30 Gy of fWTI (Fig. 5A, C). After a fractionated 24 Gy dose of WTI, the mean level of vimentin was increased 1.4, 1.8 and 2 fold at 1, 2 and 5 months postirradiation, respectively (Fig. 6C).

### WTI leads to co-localization of pro-SP-c and α-SMA protein in the alveoli

Previous analyses of human fibrotic lung tissue revealed AE2 cells with mesenchymal features, suggesting EMT could occur in patients with idiopathic pulmonary fibrotic diseases [13]. To determine if radiation can cause the transdifferentiation of AE2 cells to mesenchymal-like cells, lung tissue was harvested at 14 weeks after irradiation with a single 12 Gy dose of WTI, and the location of the pro-SP-c and α-SMA proteins in the irradiated lung tissue compared to the location in unirradiated lung tissue from age-matched controls. The two proteins co-localized in the alveoli of the irradiated lungs, but not in the alveoli of the unirradiated lungs (Fig. 6), suggesting that radiation can cause the transdifferentiation of AE2 cells to a mesenchymal-like phenotype.

### WTI leads to increased TGF-ß1 and VCAM-1 protein levels

**Figure 7:**
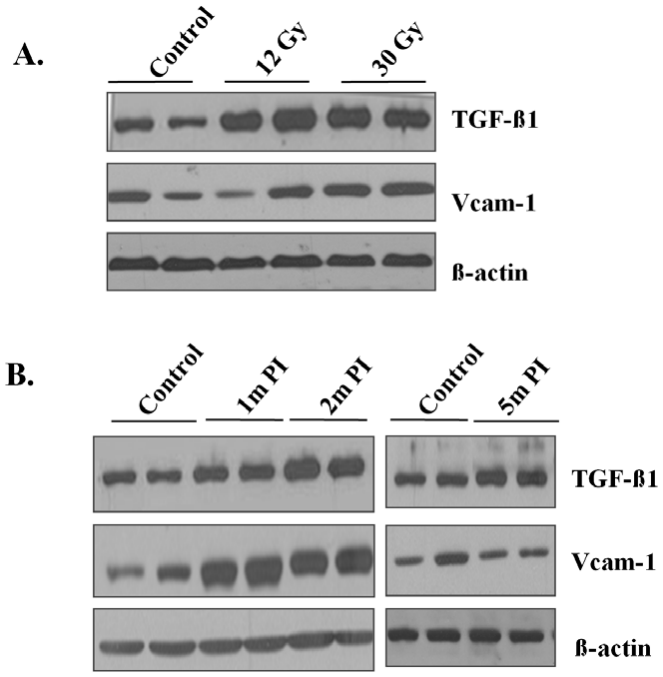
Irradiating the lungs of young adult female FVB/N mice with either a single 12 Gy dose or fractionated 24 or 30 Gy doses of ^137^Cs γ-rays leads to increase in VCAM-1 and TGF-ß1 protein in the lung. **A**: The increase in VCAM-1 and TGF-ß1 is similar at 14 weeks after a single 12 Gy dose or a fractionated 30 Gy dose. **B**: Both VCAM-1 and TGF-ß1 increase up to 2 months after a fractionated 24 Gy dose and then decrease up to 5 months postirradiation.

Levels of TGF-ß1 proteins, a fibrogenic cytokine, increased similarly at 14 weeks after irradiation with a single 12 Gy dose or a 30 Gy dose of fWTI (Fig. 7A). The TGF-ß1 protein level increased up to 2 months after 24 Gy fWTI (Fig. 7B). This was followed by a decrease in the TGF-ß1 protein level over the next 3 months that did not reach the level in the age-matched unirradiated controls. VCAM-1 is a member of the immunoglobulin superfamily with a critical role in mediating the adhesion of leukocytes to endothelial cells in various acute and chronic inflammatory diseases [31]. The VCAM-1 protein level was increased slightly at 14 weeks after irradiation with a single 12 Gy dose or a 30 Gy dose of fWTI (Fig. 7A). After 24 Gy of fWTI, the VCAM-1 protein level increased substantially up 2 months postirradiation, but returned to unirradiated control values by 5 m postirradiation (Fig. 7B).

## Discussion

Radiation-induced pneumonitis and subsequent fibrosis are major dose-limiting complications for patients receiving thoracic RT. Various mouse strains have been used to study radiation-induced normal tissue injury; their response is strain-dependent. In the current study, we demonstrated for the first time, i] the sensitivity of young adult female FVB/N mice to thoracic irradiation and ii] the involvement of EMT in radiation-induced lung injury.

In our study, irradiating the thorax of young adult female FVB/N mice with single doses of 12 and 13 Gy led to 50% mortality at 14 and 11 weeks postirradiation, respectively (Fig. 1). In contrast, Steel *et al* reported that the median survival time of C57BL mice after doses of 12–20 Gy is between 200–300 days [32]. Yang *et al* reported that ∼50% of the C57BL mice died within 32 weeks after a single 14.5 Gy dose of WTI [33]. O'Brien et al. reported that the mean survival time of C57BL mice was 21.8±1.5 weeks after a single 18 Gy dose of WTI [34]. Histological analysis of lung tissues from FVB/N mice revealed an extensive alveolar inflammation and regional fibrosis 14 weeks after a single 12 Gy dose of WTI (Fig. 2). In contrast, fibrotic lesions were observed 24 and 26 weeks postirradiation in fibrosis-prone C57/B6 mice after single 12 Gy and 12.5 Gy doses of WTI, respectively [35], [36]. These results indicate that the lungs of young adult female FVB/N mice are radiosensitive, making them an appropriate model for studying both radiation-induced pneumonitis and fibrosis, particularly since many transgenic mice are created on a FVB/N background.

One feature of radiation pneumonitis is excessive leakage of fluid from the vessels into the alveolar space, which could impede alveolar gas exchange. Aquaporin-5 has been described as playing a critical role in maintaining water permeability across the cell membrane and also in removal of pulmonary edema fluid from the alveolar space [37]. Aquaporin-5 knock-out mice exhibit a 90% decrease in airspace-capillary water permeability, suggesting an important role for aquaporin-5 in maintaining normal lung physiological function [38]. Decreased expression of aquaporin 5 has been reported in pathological conditions such as acute lung injury [39], [40] and lung fibrosis [41]. Moreover, a significant reduction in aquaporin-5 mRNA and protein level has been associated with pulmonary inflammation and edema resulting from adenoviral infection [40]. Gabazza et al. noted decreased expression of aquaporin-5 in alveolar type I cells in a mouse model of bleomycin-induced lung fibrosis; aquaporin-5 knock-out mice revealed a fibrotic phenotype [41]. Taken together, these data indicate that down-regulation of aquaporin-5 results in abnormal lung fluid metabolism in many diseases. In our study, a decrease in the aquaporin-5 protein level was measured in lungs of mice after a single 12 Gy dose or a fractionated 30 Gy dose of WTI (Fig. 3A); a time-dependent decrease in the aquaporin-5 protein level was measure after a fractionated 24 Gy dose of WTI (Fig. 3B, C). These novel findings suggest that aquaporin-5 may be an important biomarker of radiation-induced lung injury.

The lung alveolar epithelium is in direct contact with air. AE1 cells are terminally differentiated, highly susceptible to injury, and incapable of self-renewal. In contrast, AE2 cells are highly resistant to insults, able to self-renew, and serve as stem cells for producing AE1cells; AE2 cells are known as the defenders of the alveoli [42]. When the alveolar epithelium is damaged, AE2 cells start proliferating and transdifferentiate into AE1 cells to re-establish a functional alveolar epithelium. Alveolar epithelial injury followed by abnormal epithelial repair appears to be a key pathological feature of lung fibrosis. Increased proliferation/hyperplasia of AE2 cells has been frequently observed in injured and irradiated lungs [43], [44]. Pro-SP-c, a surfactant protein, is expressed only by AE2 cells; thus, it has been used as a marker of type II cell differentiation in the mammalian lung [13]. The increased number of pro-SP-c^+^ cells (Fig. 4A, B) and the increased pro-SP-c protein levels (Fig. 4C) measured after both single and fractionated doses of WTI likely reflects proliferation of AE2 cells in an attempt to repair the radiation-induced lung damage.

Myofibroblasts, α-SMA expressing fibroblasts, are a prominent source of type I collagen and fibrogenic/inflammatory cytokines in fibrotic lesions. Several origins for myofibroblasts have been proposed; resident fibroblasts seem to be the most common one. Bone marrow-derived circulating cells have also been suggested as an alternative source of myofibroblasts. Epperly et al. demonstrated that marrow-derived cells constitute 20 to 50% of the cells in radiation-induced fibrotic areas using GFP–positive bone marrow cells [45]. Consistent with these results, collagen-producing lung fibroblasts derived from bone marrow progenitor cells have been detected in fibrotic tissues in bleomycin-induced fibrosis [46]. However, these marrow-derived fibroblasts did not express α-SMA and were resistant to the fibroblast to myofibroblast conversion by TGF-ß1 [46]. Recent studies indicate that myofibroblasts can also arise from resident epithelial cells [12], [47] that undergo EMT. Liu et al. [48] reported expression of α-SMA and vimentin in kidney tubular epithelial cells in a rat model of radiation nephropathy, supporting the hypothesis that radiation leads to the transition of tubular epithelial cells to myofibroblasts. EMT and increased cell motility has also been reported in the irradiated human lung A549 cells [49]. Using an *in vitro* model, we have demonstrated that irradiation of AE2 cells resulted a transition of epithelial to a myofibroblast-like phenotype, which was mediated by the ROS/ERK/GSK-3ß/Snail pathway [50]. However, EMT in the irradiated lung has not been previously reported. In this study, a significant decrease in the protein levels of the epithelial cell markers, E-cadherin (Fig. 5) and aquaporin-5 (Fig. 3), was measured after both single and fractionated doses of WTI. This was accompanied by a concomitant increase in vimentin (Fig. 5), a mesenchymal marker. Moreover, double immunoflorescence staining showed co-localization of the pro-SP-c and α-SMA proteins in the alveoli of irradiated lungs (Fig. 6), implying that AE2 cells had gained a mesenchymal-like phenotype. Our data suggest that AE2 to mesenchymal transition occurs in the irradiated lungs of FVB/N mice. However, it is not known if other epithelial cells in the lung can differentiate into myofibroblasts. The relative contributions of epithelial cells in the irradiated lung to the overall increase in the myofibroblast population and the pathogenic role that EMT plays in radiation-induced lung fibrosis remain to be investigated.

TGF-β1 plays an important role in the development of lung fibrosis [51]. Increased TGF-β1 expression leads to recruitment of monocytes and macrophages to the inflammatory site, enhances the maturation and activation of fibroblasts, and stimulates EMT [52]. Chronic radiation-induced lung injury has been reported to increase expression and activation of TGF-β1, which leads to parenchymal cell depletion and excess fibrosis [53]. TGF-β1 expression in the plasma of patients immediately after RT has been used as an important marker to predict the risk for radiation-induced lung injury [54]. In our study, both single and fractionated doses of WTI increased the TGF-β1 protein levels in the lung (Fig. 7). The sustained increase in the TGF-ß1 protein level in the irradiated lungs of FVB/N mice further demonstrates the important role of TGF-ß1 in the pathogenesis of radiation-induced lung injury.

Recently, chromatin remodeling via posttranslational histone modification was found to function in a genome-wide manner and contributes to an extensive range of biological functions [55]. Histone deacetylases (HDACs) are known as modulators of gene transcription, which is important for cell function, proliferation and differentiation. HDAC inhibitors have been reported to induce protein hyperacetylation, chromatin remodeling, transcriptional activation and repression, cell-cycle arrest, and cell death [56], [57]. Preclinical studies indicate that HDAC inhibitors can effectively block cardiac, skin, liver and renal fibrosis [58]. *In vitro* and *in vivo* investigations indicate that HDAC inhibitors modulate fibrosis by suppressing ECM production, inhibiting myofibroblast activation, blocking EMT, and/or reducing pro-inflammatory cytokine production [11], [59]–[63]. Furthermore, recent studies suggest that topical treatment of rat skin with HDAC inhibitor 4-Phenyl butyrate has been shown to promote wound healing, reduce skin fibrosis, and decrease tumorigenesis after irradiation [64]. In light of these findings, effect of on HDAC inhibitors on radiation-induced lung damage in FVB/N mice is underway in our lab.

In summary, the current findings indicate that the lungs of female FVB/N mice are radiosensitive and represent an appropriate model for investigating radiation-induced lung inflammation and fibrosis. The marked changes in the levels of the alveolar epithelial proteins, E-cadherin, aquaporin-5, and pro-SP-c, appears to be associated with the development of radiation-induced lung injury in FVB/N mice, suggesting that these proteins may serve as sensitive indicators of radiation-induced lung damage. WTI resulted in the loss of epithelial markers and a subsequent increase in the levels of mesenchymal proteins, indicating that EMT occurs in irradiated lung tissue. Although the *in vivo* significance of EMT is unclear, radiation-induced alterations in the alveolar epithelium phenotype implicate impairment of alveolar epithelial function in producing fibrosis. Future research to identify and quantify the specific mechanisms involved in producing radiation-induced fibrosis should provide targets for the development of interventions that prevent/ameliorate this devastating complication of WTI.
